# Fragile Mental Retardation Protein Interacts with the RNA-Binding Protein Caprin1 in Neuronal RiboNucleoProtein Complexes

**DOI:** 10.1371/journal.pone.0039338

**Published:** 2012-06-21

**Authors:** Rachid El Fatimy, Sandra Tremblay, Alain Y. Dury, Samuel Solomon, Paul De Koninck, John W. Schrader, Edouard W. Khandjian

**Affiliations:** 1 Centre de Recherche, Institut Universitaire en Santé Mentale, Québec, PQ, Canada; 2 Département de Psychiatrie et de Neurosciences, Faculté de Médecine, Université Laval, Québec, PQ, Canada; 3 Département de Biochimie, Microbiologie et Bio-Informatique, Université Laval, Québec, PQ, Canada; 4 The Biomedical Research Center, University of British Columbia, Vancouver, British Columbia, Canada; University of Münster, Germany

## Abstract

Fragile X syndrome is caused by the absence of the Fragile X Mental Retardation Protein (FMRP), an RNA-binding protein. FMRP is associated with messenger RiboNucleoParticles (mRNPs) present in polyribosomes and its absence in neurons leads to alteration in synaptic plasticity as a result of translation regulation defects. The molecular mechanisms by which FMRP plays a role in translation regulation remain elusive. Using immunoprecipitation approaches with monoclonal Ab7G1-1 and a new generation of chicken antibodies, we identified Caprin1 as a novel FMRP-cellular partner. *In vivo* and *in vitro* evidence show that Caprin1 interacts with FMRP at the level of the translation machinery as well as in trafficking neuronal granules. As an RNA-binding protein, Caprin1 has in common with FMRP at least two RNA targets that have been identified as *CaMKIIα* and *Map1b* mRNAs. In view of the new concept that FMRP species bind to RNA regardless of known structural motifs, we propose that protein interactors might modulate FMRP functions.

## Introduction

Fragile X syndrome (FXS) is caused by the absence of expression of the Fragile X Mental Retardation Protein (FMRP) [1]–[3]. This RNA-binding protein widely expressed in mammalian tissues [4] is particularly abundant in neurons [5], and is a component of messenger ribonucleoprotein complexes (mRNP) associated with brain polyribosomes [6]–[8]. Its presence within the translational apparatus suggests that it is involved in control of mRNAs. In addition to its main location in neuronal cell body, FMRP is also found in growth cones and dendritic spines suggesting that it plays also a role in regulating local protein synthesis in micro-domains [3], [9]. In between the soma and these micro-domains, FMRP is found travelling in granules that contain packed mRNAs to be delivered at these micro-sites. The current concept is that absence of FMRP induces translation dysregulation and defects in mRNA transport that are thought to alter local protein synthesis essential for synaptic development and maturation [3], [10]–[12]. One of the consequences of the lack of FMRP is the presence of abnormal looking immature and supernumerary neuronal dendritic spines in the brains of fragile X patients [13], [14], that ultimately lead to mental retardation in FXS patients.

FMRP has been reported to associate with several hundred of different mRNAs as detected by co-immunoprecipitation [15], antibody positioned RNA amplification (APRA) [16] and *in vitro* by affinity capture [17] and cDNA-SELEX [18]. More recently, using high-throughput sequencing of RNAs isolated by cross-linking immunoprecipitation (HITS-CLIP), Darnell *et al.* [19] succeeded to identify those mRNAs in direct contact with FMRP that are thought to be the *bona fide* targets. In addition to its affinity to RNA, FMRP has the ability to interact with a series of proteins either directly or indirectly [20]. These interacting proteins might modulate FMRP functions by inducing structural changes in its conformation [21]. Two regions in FMRP, at the NH2- and at the COOH-termini have been reported to mediate interactions with protein partners. Interactors such as FXR1P, FXR2P, NUFIP, 82-FIP, CYFIP1 and CYFIP2 bind the NH2-terminal region (reviewed in [2]), while MSP-58, KifC3, Ran-BPM and SMN have affinities to regions situated at the C-terminus [22]–[25]. Other proteins such as nucleolin, YB-1/p50, Pur-α, myosin Va, kinesin, RNG140 and Stauffen have been detected in complexes containing FMRP either by immunoprecipitation or immunostaining approaches [26]–[29], but it is not known whether they interact physically with FMRP.

In search for new proteins that interact directly with FMRP in neurons, we identified Caprin1, an RNA-binding protein, as a novel FMRP partner. Interestingly, Caprin1 shares several features with FMRP and has been also proposed to control translation in neurons [30], [31].

## Results

### Monoclonal Antibody 7G1-1 Recognizes Two Different RNA-binding Proteins

**Figure 1:**
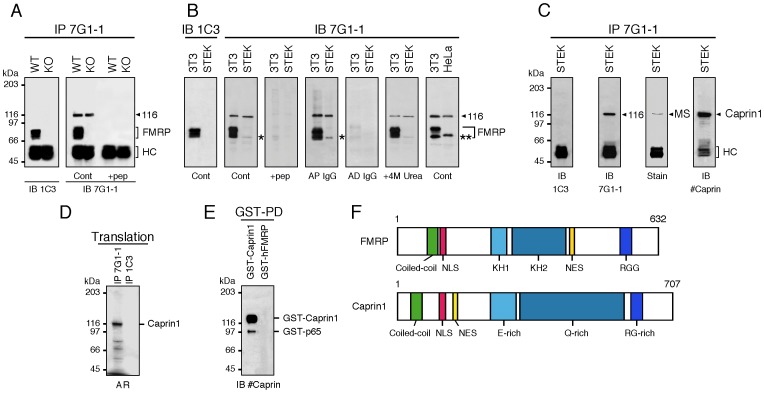
mAb7G1-1 detects mFMRP and Caprin1. **A**) Immunoprecipitation analyses of WT and KO2 mouse brain extracts with mAb7G1-1 followed by immunoblotting with mAb1C3 (left panel in **A**) or with mAb7G1-1 (right panel in **A**). In addition to mFMRP, a clear band at 116 kDa is detected in WT immunoprecipitates. A similar band is also detected in KO2 extracts. Both bands are absent when immunoprecipitation is performed in the presence of the epitope peptide KHLDTKENTHFSQPN. **B**) Immunoblot analyses with mAb7G1-1 of 3T3, STEK and HeLa cell extracts. While mAb3C1 detects only mFMRP in 3T3 extracts, mAb7G1-1 reacts with both mFMRP and p116. An additional weak band is also detected in both extracts at 65 kDa (indicated by a star). mAb7G1-1 does not react with hFMRP from HeLa extracts, but recognizes p116. Note the presence of the additional band that migrates slightly above 65 kDa in human HeLa extracts (double star). **C**) Extracts from STEK cells were immunoprecipitated with mAb7G1-1. Immunoblot analyses with mAb1C3 reveal that mFMRP is indeed absent, while mAb7G1-1 reacts with p116. The Coomassie brillant blue stained band at 116 kDa was excised and analyzed by mass spectrometry and was identified as Caprin1. Immunoblot analyses with rabbit antisera raised against hCaprin1 confirmed the nature of p116 as Caprin1. **D**) *In vitro* translated ^35^S-labeled Caprin1 is immunoprecipitated by mAb7G1-1. **E**) Recombinant GST-Caprin1 isolated on Glutathione-Sepharose beads is revealed with anti-Caprin1 IgG in immunoblot analyses. Note the presence of the minor truncated band at ∼95 kDa. **F**) Structural comparisons between FMRP and Caprin1. WT and KO: wild type C57BL/6J and *Fmr1* ^−/−^KO2 mice, respectively. IP: immunoprecipitation; IB: immunoblot; Cont: control; HC: IgG heavy chains; AP: affinity purified; AD: affinity depleted; AR: autoradiography.

In an effort to identify new proteins that interact with FMRP, we thought to isolated the complex containing FMRP using an immunoprecipitation approach. Brain lysates prepared from WT and from *Fmr1* ^−/−^ KO2 mice [32] were immunoprecipitated with mAb7G1-1 [15], [33] under conditions as close as possible to those described [15] and the retained material analyzed by immunoblotting using mAb1C3. In agreement with Brown *et al.* [15], we detected mFMRP in immunoprecipitates from wild type C57BL/6J brain, while, as expected, no corresponding signals were present in extracts from *Fmr1* ^−/−^ KO2 mice (Figure 1A). However, when mAb1C3 was substituted by mAb7G1-1 for immunoblotting, a clear additional band at ≈116 kDa was constantly observed (Figure 1A). Surprisingly, this band was also present in immunoprecipitates from brain extracts prepared from the *Fmr1* ^−/−^ KO2 mice. Pre-incubation of mAb7G1-1 with the epitope peptide corresponding to mFMRP 354-KHLDTKENTHFSQPN-368 was effective in completely inhibiting its immunoprecipitation (Figure 1A) as previously reported [15]. Unexpectedly, we also observed that preincubation of mAb7G1-1 with the peptide resulted in the disappearance of the 116 kDa band, even in the case of the *Fmr1* ^−/−^ KO2 extracts.

Having shown that mAb7G1-1 immunoprecipitates both mFMRP and the unknown protein p116, we further tested the behaviour of mAb7G1-1 by immunoblot analyses. Using mAb1C3 for immunoblotting, the expected picture (Figure 1B) of mFMRP and its isoforms was revealed in 3T3 cells. In contrast, no signals could be observed in STEK cells devoid of FMRP [34]. On the other hand, in immunoblotting with mAb7G1-1, the antibody clearly reacted with both mFMRP and p116 in 3T3 extracts, and only with p116 in STEK extracts. In repeated analyses, an additional band was also detected at ∼65 kDa (Figure 1B indicated by a star). This peptide corresponds to a cleavage product of p116 as shown below. All signals were abolished when the hybridoma supernatant was pretreated with the epitope peptide, as was the case for the immunoprecipitation analyses (see above). Recognition of both mFMRP and p116 by mAb7G1-1 was resistant to washes in 4 M urea (Figure 1B), indicating its strong affinity to both protein targets. To rule out for the presence of a contaminating antibody, we affinity-purified the anti-mFMRP using recombinant mFMRP and the resulting purified IgG still reacted with both proteins, while no signal could be revealed using the IgG depleted supernatants. Identical results were obtained using purified 7G1-1 obtained from Jennifer Darnell (results not shown). Finally, total extracts from 3T3 and HeLa cells were also analyzed. The rational for using HeLa extracts was that mAb7G1-1 has been reported not to react with hFMRP [15]. As shown in Figure 1B, indeed mAb7G1-1 does not pick up hFMRP, however the p116 signal still remained. We noted that the migration of the 65 kDa band in human cells was slightly slower compared to mouse (double star), as also observed for normal and X-fragile human lymphoblastoid cells (data not shown).

Although 7G1-1 is a monoclonal antibody, we hypothesized that in addition to mFMRP, it could cross-react with a yet unknown protein. To test this hypothesis, extracts prepared from STEK cells were immunoprecipitated with mAb7G1-1 and the eluted material analyzed by immunoblotting. As expected no mFMRP band could be detected after reaction with mAb1C3 while, again a clear and single band of approximately 116 kDa was visualized after reaction with mAb7G1-1 (Figure 1C). A band of similar apparent molecular weight was present after Coomassie brilliant blue staining of a SDS-PAGE. To identify this protein, the stained band was excised from the gel and submitted to mass spectrometry. Using the Scaffold 3 search program, the 22 trypsin generated polypeptides were shown to correspond to mouse Caprin1 (Cytoplasmic activation- and proliferation-associated protein 1) [30]. To confirm the MS identification, two different anti-sera directed against Caprin1 were used and both strongly reacted with p116 in immunoblot analyses.

To further confirm that p116 is Caprin1, *in vitro* translated ^35^S-labeled Caprin1 was immunoprecipitated with mAb7G1-1 and analyzed by SDS-PAGE. Autoradiography of the dried gel revealed a strong labeled band at 116 kDa while no signals were observed in immunoprecipitates with mAb1C3 (Figure 1D). In addition to the major 116 kDa signal, several bands were detected around 60–70 kDa corresponding to *in vitro* synthesized truncated Caprin1 forms (see below). However, using this approach, it was not possible to determine whether the p65 form shown in Figure 1B corresponded to a Caprin1 truncated form or to a normal isoform. We therefore prepared recombinant GST-Caprin1 that was used in an affinity assay with Glutathione-Sepharose beads. The retained material was eluted and analyzed by immunoblotting using anti-Caprin1 IgG. The results showed clearly that the major band detected around 140 kDa corresponded to GST-Caprin1 and that the minor band around 95 kDa to GST-p65 (Figure 1E). These results indicate that p65 is a truncated form of Caprin1 and does not arise from a spliced variant since *in vitro* translated and bacterial recombinant GST-Caprin1 were used in these assays.

One possible explanation of the recognition of both mFMRP and Caprin1 by mAb7G1-1 would have been that the original hybridoma 7G1-1 secreting cells were contaminated by an anti-Caprin1 hybridoma. In an attempt to purify the anti-FMRP hybridoma, we subcloned the original 7G1-1 cells and obtained 14 single-cell colonies. All subclones secreted antibodies of IgG2b type (as for the original clone confirmed by the Developmental Studies Hybridoma Bank, University of Iowa, USA) that reacted simultaneously with both mFMRP and Caprin1.

### Caprin1 is a Novel mFMRP-interacting Protein

The results reported above clearly demonstrate that mAb7G1-1 reacts simultaneously with two different proteins, namely FMRP and Caprin1. While it is not exceptional that monoclonal antibodies might not be exclusive to a single protein, the present situation was intriguing. Indeed Costa *et al*. [35] have reported in *Drosophila* that immunoprecipitates of Orb, the ortholog of the vertebrate CPEB1 (Cytoplasmic Polyadenylation Element Binding protein-1), contain dFMRP, plus the ortholog of Caprin1 and Rin, the *Drosphila* ortholog of the vertebrate RNA-binding protein G3BP-1, the heterodimeric partner of Caprin1 in vertebrates. Similarly to FMRP, Caprin1 is an RNA-binding protein that is highly expressed in brain and is thought to play a role in local translation control in neurons [30], [31]. In addition to these features, Caprin1 shares with FMRP a series of similar characteristics such as a coiled-coil domain, a nuclear localization signal (NLS), a nuclear export signal (NES), an E-, a Q- rich regions that are present in RNA-binding proteins [36], [37]. Finally, an RG-rich region is present at the C-terminus of the protein (Figure 1F).

Altogether these intriguing observations prompted us to further investigate whether Caprin1 is a new FMRP interactor.

#### a) Immunoprecipitation studies

Based on the results reported above, we concluded that the mAb7G1-1 was not a reliable tool to immunoprecipitate FMRP to study its associated proteins. We therefore decided to obtain new antibodies to FMRP in chicken, since due to the phylogenetic distance between birds and mammals, there are more antigenic differences between mammal and chicken FMRP, and thus chickens would make more antibodies against many non conserved epitopes. His6X-recombinant hFMRP was prepared and injected in a series of chickens and the IgY isolated from eggs. Of the several preparations of IgY obtained, #C10 was retained and used throughout the present study.

**Figure 2:**
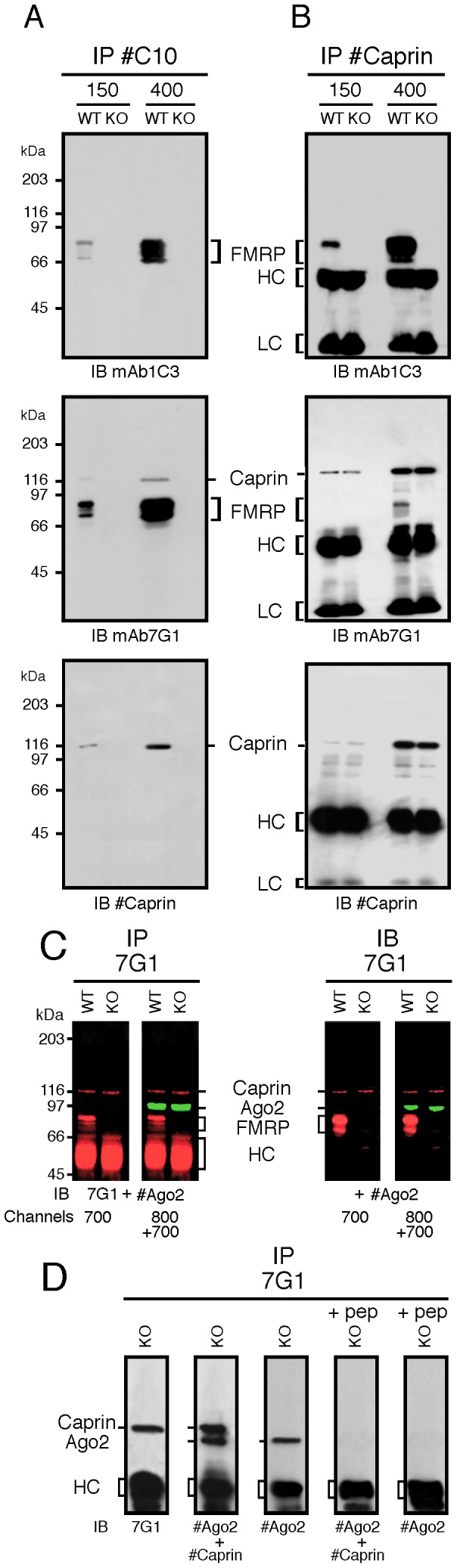
mFMRP co-immunoprecipitates with Caprin1. Total brain extracts from WT and KO mice were subjected to immunoprecipitation with IgY#C10 (**A**) and anti-Caprin1 IgG (**B**) and the eluted proteins were analyzed by immunoblotting using mAb1C3, mAb7G1 and anti-Caprin1 IgG. Note that a high salt concentration was necessary to immunoprecipitate mFMRP in association with Caprin1. **C**) mAb7G1-1 has no anti-Ago2 intrinsic activity, however Ago2 is co-immunoprecitating with Caprin1 (left panels) as detected using the Odyssey Infrared Imaging System. In the right panels are shown the results proving that mAb7G1-1 does not possess an anti-Ago2 activity as detected by direct immunoblot analyses. Membranes were either scanned at 700 nm or simultaneously at 700 plus 800 nm. **D**) The epitope peptide (see Figure 1A) abolishes co-precipitation of Ago2 with Caprin1.

To test whether IgY#C10 effectively immunoprecipitates FMRP, whole brain lysates were prepared from WT and from *Fmr1* KO2 mice and subjected to immunoprecipitation under different conditions. The results showed that a high salt condition was required to quantitatively immunoprecipitate mFMRP in agreement with Brown *et al.* [15]. A 15–20 fold increase in the recovery of immunoprecipitated mFMRP was observed when lysates were treated at 400 mM NaCl as compared to 150 mM NaCl (Figure 2A). However, no difference in mFMRP recovery was observed whether the lysates were treated with EDTA or MgCl_2_ (not shown). When immunoprecipitates obtained with IgY#C10 were tested in immunoblots with mAb7G1-1, mFMRP was detected in WT extracts together with Caprin1. This was further confirmed by immunoblotting with an antiserum directed against Caprin1 that revealed the p116 band. To confirm that Caprin1 co-immunoprecipitates with mFMRP, the reverse immunoprecipitation was carried out with an anti-Caprin1 antibody. Using the same sequence of analyses applied to the immunoprecipitated materials with IgY#C10, mFMRP was detected confirming the presence of the two proteins (Figure 2B).

It has been reported previously that certain batches of mAb7G1-1 immunoprecipitate, in addition to FMRP, two other proteins [38]. These proteins were identified as Ago2 and p137, and the authors concluded that mAb7G1-1 carries an anti-Ago2 activity. First, it should be recalled that p137 glycosylphosphatidylinositol-anchored membrane protein is in fact Caprin1 (http://www.genecards.org/cgi-bin/carddisp.pl?gene=CAPRIN1; see also ref. 30). Having resolved the first part of the puzzle, we then wished to determine whether mAb7G1-1 has an additional intrinsic anti-Ago2 activity. Immunoprecipitated proteins obtained from WT and KO brain lysates were analyzed simultaneously for the presence of FMRP, Caprin1 and Ago2 on a single membrane, using the Odyssey Infrared Imaging System from LI-COR (Figure 2C). mAb7G1-1 was revealed with secondary antibodies IR-Dye 700 (red) and rabbit antibodies against Ago2 with IR-Dye 800 (green). Using the 700 nm single channel detecting the red fluorescence emission, we observed that mAb7G1-1 immunoprecipitates only FMRP and Caprin1, in agreement with the results presented in Figure 1A. Using simultaneously the two channels 700 and 800 nm, an additional band could be revealed in green at ≈ 95 kDa corresponding to Ago2 (Figure 2C). These results clearly indicate that Ago2 is present in immunoprecipitates obtained with mAb7G1-1, however the absence of red signals at 95 kDa rules out the possibility that mAb7G1-1 has an anti-Ago2 activity. In addition, direct immunoblot analyses with mAb7G1-1 using the single channel 7000, revealed only 2 bands corresponding to Caprin1 and FMRP, while Ago2 had to be detected by an additional anti-Ago2 antibody shown in green at channel 800 (Figure 2C). These analyses prove that mAb7G1-1 does not carry an anti-Ago2 activity and indicate that the latter is co-precipitating with Caprin1. In a last set of analyses (Figure 2D), Ago2 signals co-precipitating with Caprin1 were abolished when the immunoprecipitations were conducted in the presence of the epitope peptide (see Figure 1A) indicating that Ago2 precipitation is dependent on Caprin1. Altogether, these results indicate that Ago2 is present in a complex containing Caprin1, however it is not known whether the two proteins intercact physically. Work is in progress to determine whether Caprin1, and at the same time FMRP, binds to Ago2 directly or indirectly.

#### b) Mapping of Caprin1-FMRP interactions

**Figure 3:**
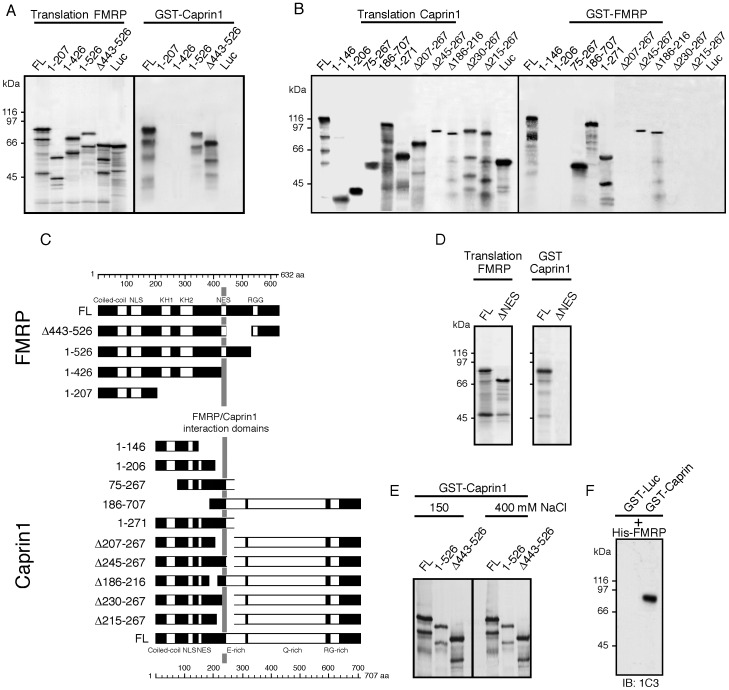
Mapping of interaction between hFMRP and hCaprin1 in pulldown assays. A ) Pulldown assay using 1 µg of the fusion protein GST-Caprin1 immobilized on beads and *in vivo* translated ^35^S-labelled full length FMRP (FL) and its truncated and deleted versions. **B**) Reverse pulldown assay using immobilized GST-FMRP incubated with ^35^S-labeled full length Caprin1 (FL) and its truncated and deleted versions. In both cases, ^35^S-labeled Luciferase (Luc) was used as a negative control. **C**) Schematic diagram summarizing the data presented in **A** and **B**. **D**) Refine region in FMRP NES (amino acids residues 424–440) required for binding to Caprin1. **E**) The interaction of FMRP with Caprin1 is stable in the presence of 400 mM NaCl. **F**) The interaction of Caprin1 and FMRP is direct in a pulldown assay using recombinant proteins.

To verify whether FMRP interacts physically with Caprin1, we performed a series of pull-down assays using hFMRP and hCaprin1 full-length proteins, as well as their truncated and deleted forms. These proteins were produced and labeled with [^35^S-methionine] by *in vitro* transcription-translation using the rabbit reticulocyte lysate. Full length hFMRP and hCaprin1 were produced in bacteria as glutathione *S*-transferase (GST) fusion proteins and immobilized on glutathione-Sepharose beads. Immobilized GST-hCaprin1 was incubated with either *in vitro* translated hFMRP and its truncated and deleted versions, or with luciferase as a negative control (Figure 3A). In parallel, immobilized GST-hFMRP was incubated with *in vitro* translated Caprin1 and its truncated and deleted versions (Figure 3B). The results of these pull-down assays show that the FMRP region spanning amino acids 427–442 is necessary for binding to Caprin1 (Figure 3C). On the other hand, Caprin1 region at amino acids 231–245 is necessary for interaction with FMRP. To ascertain that residues 427–442 in FMRP are involved in the mutual interaction, we tested whether FMRP lacking the NES domain (Δ423–441) binds to Caprin1. The results presented in Figure 3D clearly indicate that amino acids 424–440 are required for Caprin1 recognition.

To verify that the interaction between FMRP and Caprin1 was direct and not RNA-dependant, all GST-pulldown assays were also performed in the presence of RNase A. This treatment did not alter the results presented above, indicating that the interaction occurs at the protein-protein level (data not shown). We also tested the interaction of hFMRP with GST-Caprin1 at 400 mM NaCl to mimic the conditions used in the immunoprecipitation assays described above (see Figure 2) and observed that the *in vitro* interaction at high salt was not altered (Figure 3E). Finally, to rule out the possibility that the interaction is mediated by a third factor present in the rabbit reticulocyte lysate, we used a pull-down assay based on bacterial recombinant proteins. Recombinant His-mFMRP was incubated with glutathione-Sepharose beads loaded either with GST-Luciferase or with GST-hCaprin1. After washing in physiological buffer containing 0.1% SDS, the material bound to the beads was desorbed and analyzed by immunoblotting using mAb1C1 against FMRP. The results presented in Figure 3 F clearly indicate that the interaction of FMRP and Caprin1 is direct.

### mFMRP Co-sediments with Caprin1 in Polyribosomes and Co-localizes in Trafficking Granules

#### a) mFMRP and Caprin1 are present in polyribosomal mRNP complexes

**Figure 4:**
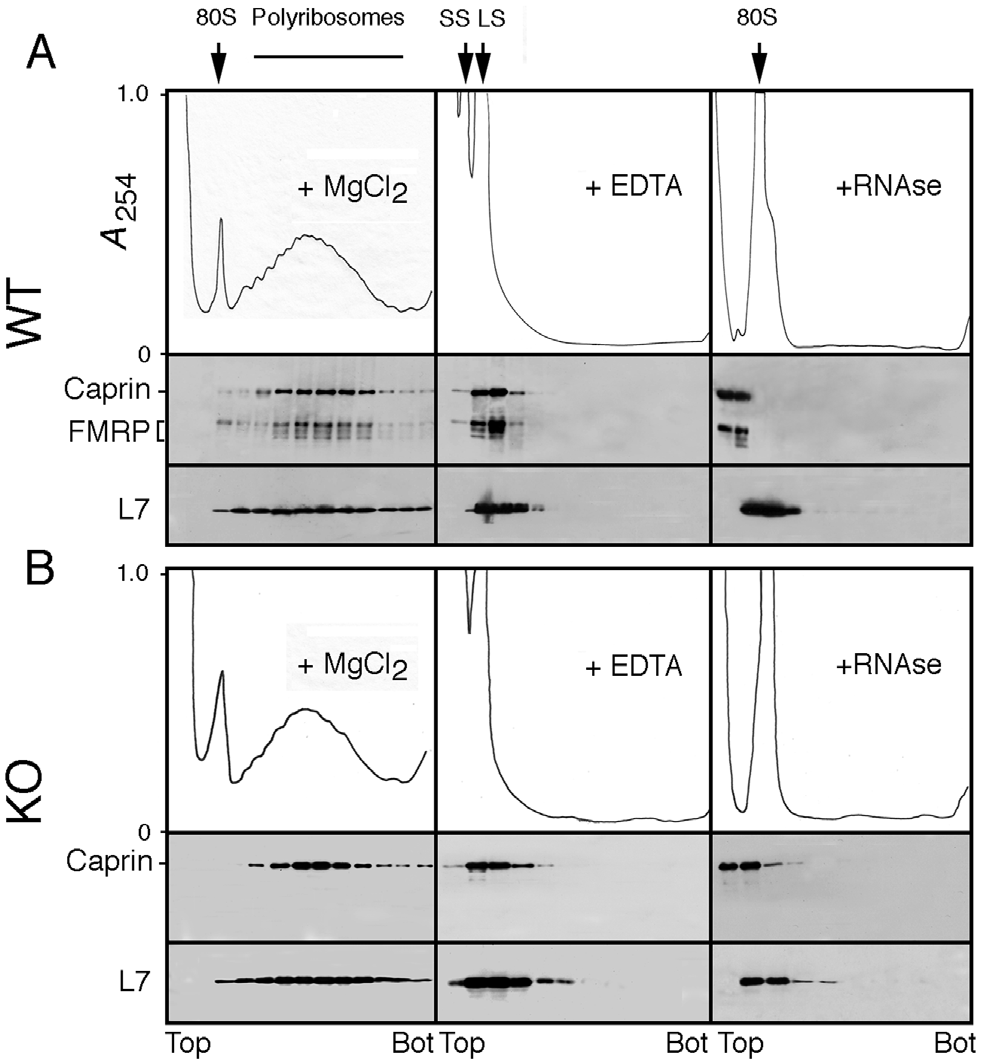
Caprin1 co-sediments with mFMRP in polyribosomes prepared from total brain and mFMRP is not required for Caprin1 to associate with polyribosomes. Aliquots containing 10–12 OD at 260 nm were analyzed by sedimentation velocity through sucrose density gradients in the presence of MgCl_2_, after incubation with 30 mM EDTA or after treatment with 100 µg/ml RNase A. mFMRP and Caprin1 were revealed simultaneously with mAb7G1-1. 80S : monosomes ; SS : ribosomal small sub-unit ; LS : ribosomal large sub-unit ; L7 : ribosomal large protein 7.

It is well established that FMRP is present in mRNP complexes associated with heavy sedimenting polyribosomes prepared from total brain [7], [8]. Since Caprin1 is strongly expressed in brain and has been reported to be a complement of the translation machinery [30], [31] we asked whether its distribution is similar to mFMRP. Brain polyribosomes were prepared as previously described and analyzed by velocity sedimentation through sucrose density gradients. In the presence of Mg^2+^, Caprin1 was detected in fractions corresponding to heavy sedimenting polyribosomes, and its distribution along the gradient mirrors that of FMRP (Figure 4). In the presence of EDTA that dissociates ribosomes into their subunits concomitant with the release of free mRNP complexes, Caprin1 as well as FMRP were detected sedimenting in the same fractions. Treatments with RNase A resulted in the complete destruction of polyribosomes and mFMRP as well as Caprin1 were displaced to the top fractions of the gradient. The presence of Caprin1 in polyribosome fractions was not altered when cytoplasmic extracts derived from KO brains were analysed, indicating that FMRP is not required for the presence of Caprin1 with polyribosomal RNPs.

#### b) Caprin co-localizes with mFMRP in neuronal cell body and in granules throughout the dendritic branching

**Figure 5:**
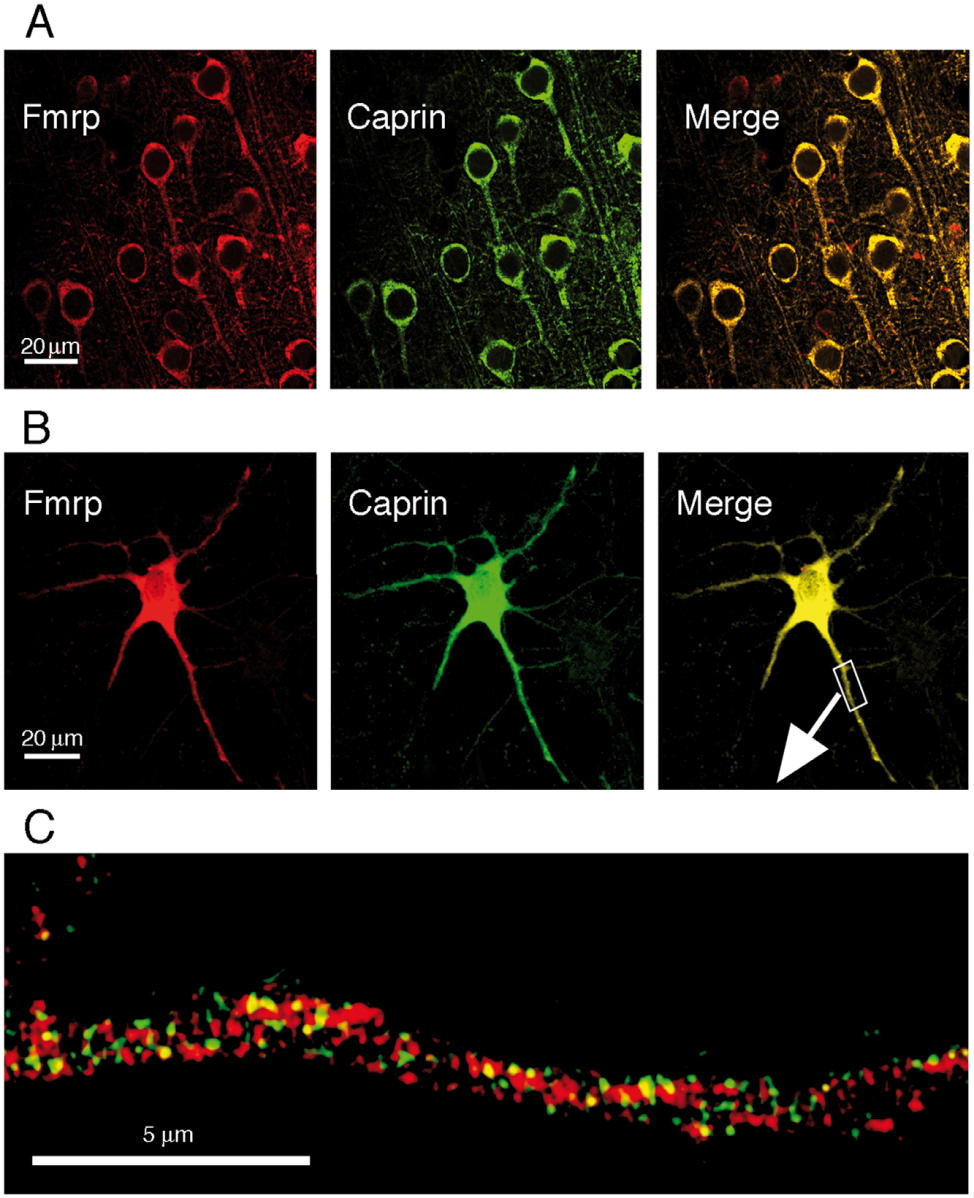
FMRP and Caprin1 partially colocalize in somato-dendritic compartments of neurons. **A**) Immunostainings of mouse brain cortical sections were carried out using anti-FMRP IgY#C10 (red) and anti-caprin1 IgG (green). FMRP and Caprin1 colocalize both in cell bodies and in axons of cortical neurons. **B**) FMRP and Caprin1 colocalize in primary cultured hippocampal neurons. Note that to illustrate staining in neuritis, a high gain was required resulting in saturation of the fluorescent signal in the cell body. **C**) The dendritic area from the box in (**B**) at higher magnification reveals that only a fraction of FMRP co-localizes with Caprin1. In this selected region, after deconvolution of the high definition images, a total of 453 granules were counted, and colocalization of mFMRP and Caprin1 accounted for only 14%.

To validate *in vivo* the potential interaction between Caprin1 and FMRP, we performed immunohistochemical double staining on mouse brain cortical sections using IgY#C10 antibody against FMRP and anti-Caprin1 IgG. Both Caprin (green) and FMRP (red) labellings were mainly cytoplasmic (Figure 5A), with a weaker granular signal in the extensions and seemed to co-localize in both the cell body and neurites.

In order to characterize with more precision the co-localization of Caprin1 and FMRP in the dendritic branching of neurons, we double-stained primary cultured rat hippocampal neurons with IgY#C10 and Caprin1 antibodies. Confocal microscopy showed a very similar distribution of FMRP and Caprin1 signals. Both proteins were strongly present in the cell body, and also present in a granular-like pattern in the dendritic arborization (Figure 5B). However, at higher magnification, and after deconvolution of the images, co-localization in neurites appeared weaker than first thought. The two proteins seemed to be present in a sub-population of granules distal from the soma (Figure 5C). Binary merging analyses using the *MetaMorph*® *Software* showed a great range of co-localizations ranging from 4 to 51% over 22 regions analyzed. Altogether, 23% of Caprin1 and FMRP signals co-localized in neurites (n = 23 neurites, and approximately 3000 granules), however this co-localization seemed highly variable from one neuron to another.

Theses observation illustrate that FMRP and Caprin1 co-localize in a granular-like pattern in neurites, even though this co-localization appears to vary across neurons. Moreover, they indicate that the composition of a given cargo may vary considerably.

### mFMRP and Caprin1 Interactions with Polyribosomes at High Salts

A requirement to quantitatively immunoprecipitate mFMRP is the use of high concentration of salt (400 mM NaCl). It is possible that the epitopes recognized by either mAb7G1-1 or IgY#C10 are masked and have to be exposed. However, it is not known whether the high salt regime has any incidence on the sedimentation properties of FMRP and Caprin1.

#### a) Sucrose gradient sedimentations

**Figure 6:**
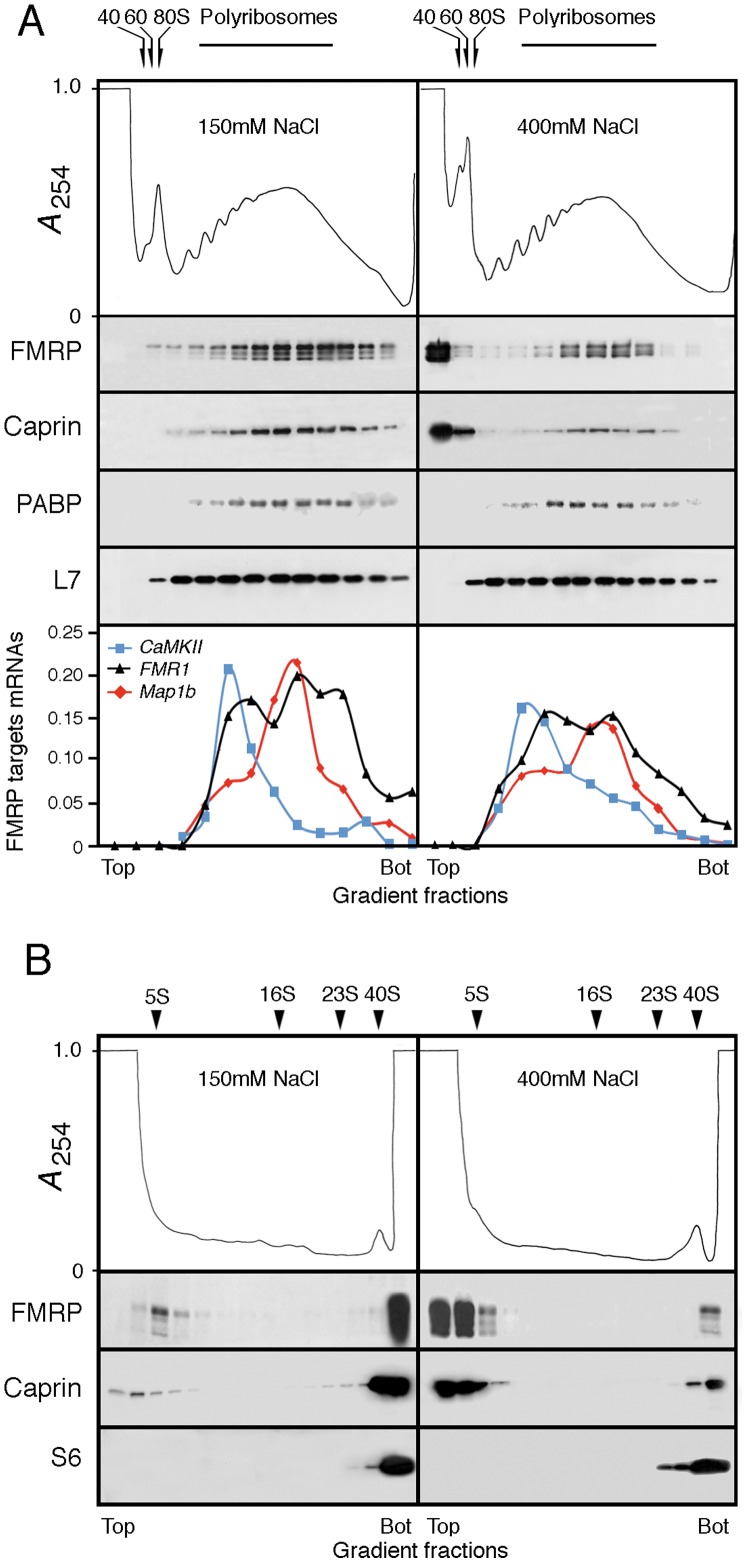
Sedimentation of FMRP and Caprin1 in presence of NaCl at physiological and at high salt conditions. A ) In presence of 150 mM NaCl, FMRP and Caprin1 are detected associated with polyribosomes. At 400 mM, a clear displacement of FMRP and Caprin1 towards the top of the gradient is observed, while PABP and the ribosomal L7 protein are not affected. In the bottom panels of **A**) are shown the distribution in the sucrose gradients of *CaMKIIα*, *Map1b* and *Fmr1,* FMRP-mRNA targets in the presence of 150 and of 400 mM NaCl. Note the slight shift toward the lighter fraction in the 400 mM NaCl condition and the absence of mRNA in the fraction containing the highest amount of FMRP at the top of the gradient. The results are presented as percent of the specific mRNA in each fraction. **B**) In presence of 400 mM NaCl, the majority of FMRP and Caprin1 are found as “floating” species in the loading volume that did not penetrate the gradient even after prolonged ultracentrifugation for 23 hrs. The position of the small ribosomal subunit was determined according to the UV profile and immunoblotting with anti-S6 protein. S values were determined using purified total RNA from *E.coli*.

Polyribosomes were prepared in a standard salt condition at 150 mM NaCl and also at 400 mM NaCl and analyzed by sedimentation through sucrose density gradients. In repeated experiments, we observed that the UV profiles obtained from the two conditions were similar but not identical, with a slight shift toward the top of the gradient in the case of the high salt concentration (Figure 6A). At 400 mM NaCl, the 80S monosome was drastically reduced, while the peaks corresponding to the 40 and 60S ribosomal subunits were more pronounced, a phenomenon known since the 70’s corresponding to the dissociation of 80S monomers that do not contain mRNA, referred to as “vacant 80S” [39]–[41]. Immunoblot analyses of the collected fractions revealed drastically reduced signals of FMRP and Caprin1 at the level of polyribosomes, while strong signals accounting for approximately 50–70% of FMRP and Caprin1 were detected at the top of the sucrose gradients. The same phenomenon was also observed for FXR1P and FXR2P (see Figure S1). In contrast, the association of PABP with polyribosomes did not show alteration at high salts as previously shown [42] and the distribution of the ribosomal L7 protein followed the UV profile. We hypothesized that FMRP and Caprin1 present in the two fractions at the top of the gradient could correspond to molecules that are not associated with mRNAs. We therefore analyzed the distribution of the three known FMRP-mRNA targets, *Fmr1, CaMKIIα* and *Map1b* along the sucrose gradient under physiological and high salt conditions. RNA was extracted from each fraction, purified, reversed transcribed and amplified by qPCR. The results presented in Figure 6A, representative of two independent gradients, clearly show that the three FMRP-targets mRNAs tested were all distributed along the polyribosomal fractions and that only a shift toward the top of the gradient (one fraction) was observed in the presence of 400 mM NaCl, as was the case for the UV profile. In repeated analyses we observed no correlation between the presence of FMRP and mRNA targets in the top fractions. To show that FMRP molecules that have been stripped from polyribosomes under the stringent conditions of high salt concentrations, were not associated with sedimenting structures, ultracentrifugation of polyribosomes was extended to 23 hours. Under these conditions, the 40S small ribosomal subunit was detected just above the bottom of the gradient. At 150 mM NaCl condition, trace amounts of FMRP and of Caprin1 could be detected around 4–5 S resulting probably from released molecules during polyribosomes extraction and manipulations. On the other hand, after treatment of polyribosomes with 400 mM NaCl, substantial amounts of FMRP and Caprin1 were detected in the two fractions at the top of the gradient corresponding to the loaded volume of the sample that did not penetrate the gradient and with an S value estimated to be less than 5S.

These results clearly indicate that a substantial amount of FMRP isolated by immunoprecipitation at high salt regime comes from molecules that are not associated with mRNAs.

#### b) Mass spectrometry analyses

**Figure 7:**
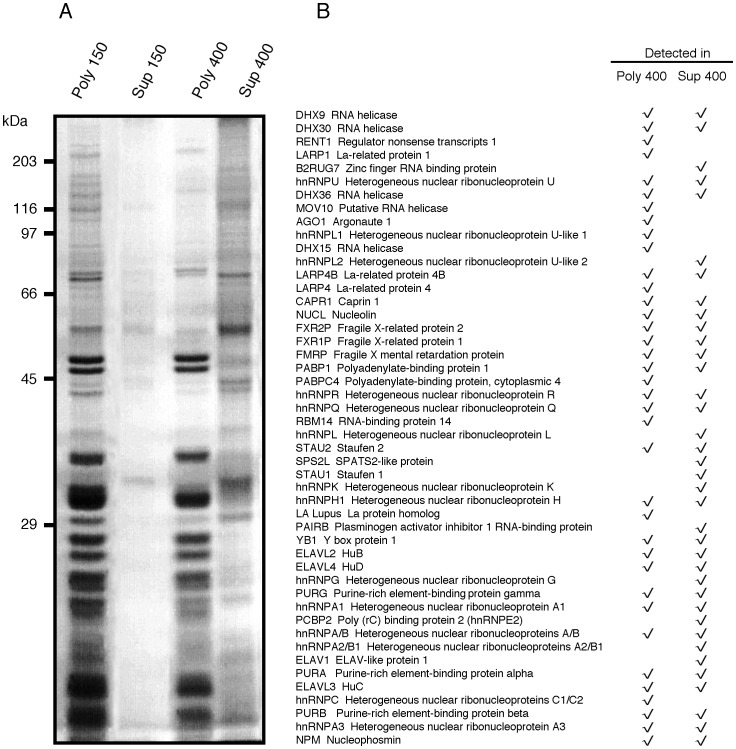
Distribution of RNA-binding proteins in polyribosomes and in salt washes. **A**) Equivalent volume aliquots from polyribosomes and from the 150 and 400 mM NaCl washes were analyzed by SDS-PAGE and the separated proteins stained with Coomassie blue. **B**) Identification by Mass Spectrometry of RNA-binding proteins present or released from polyribosomes after 400 mM NaCl washes. Poly 400: polyribosomes resistant to 400 mM NaCl; Sup 400: supernatant of the polyribosomes treated with 400 mM NaCl.

In view of the altered sedimentation properties of mFMRP at 400 mM NaCl, we wondered whether this observation was a generalized phenomenon that affected different RNA-binding proteins. Concentrated polyribosomes were washed with buffers containing either 150 or 400 mM NaCl and after 15 minutes incubation at 4°C, the samples were recentrifuged at 54,000 rpm (392,540 g) to pellet the polyribosomes (see Methods section). The pellets were resuspended in volumes equal to that of the supernatants, and aliquots of equal volume were analyzed by SDS-PAGE and the separated proteins stained with Coomassie brilliant blue. In the 150 mM NaCl washes, no significant amount of protein was detected, while at 400 mM NaCl, approximately 15% of proteins were removed from polyribosomes as calculated after scanning of the stained gels. To identify the nature of the major polypeptide bands removed from polyribosomes at 400 mM NaCl, aliquots were analyzed by SDS-PAGE and the gels cut into 20 sections that were subjected to trypsin digestion and to analyses by Mass Spectometry. Shown in Figure 7 is the distribution of identified RNA-binding proteins still associated with polyribosomes and those removed at 400 mM NaCl. These results confirm the data presented in Figure 6 (and Figure S1) as far as they concern the distribution of FMRP, FXR1P, FXR2P, Caprin1 and PABP. Interestingly, the majority of RNA-binding proteins were not equally distributed in the two fractions, for instance RENT1, Ago1, LARP1 and DHX15 were exclusively present in the polyribosomal fraction resistant to 400 mM NaCl, while hnRNPL2, Stau1, and PAIRB were found in the salt fraction. Other proteins were distributed in both fractions. As we had not the intention to establish a catalog of soluble proteins, these results should be considered as indicative. Nonetheless, they confirm the results presented in Figure 6 obtained after sedimentation in sucrose density gradients under high salt concentrations. Given the high number of heterogenous nuclear RNP proteins present in polyribosomal preparations, we wonder whether this fraction prepared according to our conditions could be contaminated by proteins that were released from nuclei during brain homogeneization. Immunoblot control analyses performed on the polyribosomal fractions with antibodies to histone H3 and H2b as well as for RNA-polymerase II indicate that this was not the case (data not shown).

### mFMRP and Caprin1 Neuronal mRNA Targets

**Figure 8:**
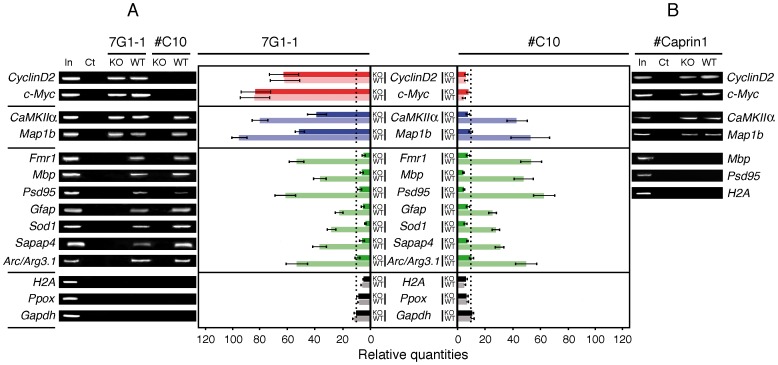
mRNAs associated with Caprin1 and FMRP. A ) mRNAs co-immunoprecipitated by mAb7G1-1 and IgY#C10 from WT and KO brain extracts were analyzed by RT-PCR and visualized by agarose gels (left panels) and by Light Cycler RT-PCR (right panels). Bars in red refer to mRNA targets to Caprin1, in blue common to Caprin1 and FMRP, in green to FMRP and in black to non-targets mRNAs. Dark colors refer to WT and pale to KO, respectively. N = 5, P≤0.001 of a pool of 10 adult brains. Vertical black dot lines represent thresholds corresponding to background. **B**) RT-PCR analyses of selected mRNAs co-immunoprecipitated with Caprin1.

In view of the results obtained with mAb7G1-1 that immunoprecipitates mFMRP and Caprin1, both RNA-binding proteins, we analyzed a restricted number of mRNAs that have been reported to be FMRP targets. Among the hundreds of mRNA obtained either by immunoprecipitation or by AMPRA, we restricted our choice to few that have been validated such as *CaMKIIα, Map1b, Fmr1, Mbp, Psd95, Gfap, Sod1, Sapap4,* and *Arc/Arg3.1* [3], [15], [16]. In addition, we also tested for *CyclinD2* and *c-Myc* mRNAs that have been reported to be Caprin1’s targets [43] while *H2A, Ppox* and *Gapdh* mRNA were used as controls. In parallel, we also immunoprecipitated FMRP with IgY#C10 that do not react with Caprin1. RNA extracted and purified from immunoprecipitated WT and KO2 brain lysates was amplified by RT-PCR with mRNA-specific primers (Table 1). Total RNA from brain was used as template for RT-PCR for each tested immunoprecipitated RNA. Consistent with our suspicions, we observed after agarose gel electrophoresis and staining with ethidium bromide that *Cyclin D2* and *c-Myc* mRNA, two mRNAs that have not been reported as FMRP targets were both immunoprecipitated with mAb7G1-1 from WT as well as from KO2 brain extracts (Figure 8A). In contrast, no signals were observed using IgY#C10. The two next tested *CaMKIIα* and *Map1b* mRNAs showed also a complex distribution, since they were detected with mAb7G1-1 in both WT and KO2 immunoprecipitates. On the other hand, using IgY#C10, *CaMKIIα* and *Map1b* were present only in WT immunoprecipitates. *Fmr1, Mbp, Psd95, Gfap, Sod1, Sapap4* and *Arc/Arg3.1* mRNAs seemed to be specifically associated with FMRP since mAb7G1-1 and IgY#C10 yielded similar signals. In parallel, we performed quantitative Light Cycler RT-PCR, which showed similar results, however we noted that *CaMKIIα* and *Map1b* mRNAs immunoprecipitated with IgY#C10 account for half values obtained with mAb7G1-1 demonstrating that both FMRP and Caprin1 interact with these targets. Finally, although the aim of these studies was not to analyze Caprin’s targets, we tested the presence of *Cyclin D2, c-Myc, CaMKIIα* and *Map1b* in immunoprecipitates with rabbit antibodies against Caprin1, which confirmed the results obtained with mAb7G1-1 using KO2 extracts (Figure 8B). On the other hand, FMRP targets *Mbp, Psd95* were not detected, as for *H2A* mRNA.

**Table 1:** Oligonucleotides primers used in RT-PCR analyses.

mRNA	Primers
*Sapap4*	F: 5′-GGAAGGCTGGTGCTGCCAGATGG-3′ R: 5′-GGGACATAAATCTCGATGCTGTC-3′
*Arc/Arg3.1*	F: 5′-TGAGACCAGTTCCACTGATG-3′ R: 5′-CTCCAGGGTCTCCCTAGTCC-3′
*CaMKIIα*	F: 5′-AATGGCAGATCGTCCACTTC-3′ R: 5′-ATGAGAGGTGCCCTCAACAC-3′
*Psd-95*	F: 5′-GTGGGCGGCGAGGATGGTGAA-3′ R: 5′-CCGCCGTTTGCTGGGAATGAA-3′
*Fmr1*	F: 5′-GACAAGTCAGGAGTTGTGAGG-3′ R: 5′-CTTTAAATAGTTCAGGTGATAATC-3′
*Cyclin D2*	F: 5′-GTGGACCCGGTCCGCAGGGC-3′ R: 5′-CCAGTTCCCACTCCAGCAGCTCC-3′
*c-Myc*	F: 5′-CTGGTCCTCAAGAGGTGCCACG-3′ R: 5′-GGGATCTGGTCACGCAGGGCAA-3′
*Map1b*	F: 5′-CTCCATCCTGCTCACCCACATTG-3′ R: 5′-GCATAAAATACTGCATTTCCTTG-3′
*Gfap*	F: 5′-CACGAACGAGTCCCTAGAGC-3′ R: 5′-TCACATCACCACGTCCTTGT-3′
*Mbp*	F: 5′-ATAACCATTCCCTGCCTCC-3′ R: 5′-TCAACCATCACCTGCCTTC-3′
*Sod1*	F: 5′-GCAGGACCTCATTTTAATCCTCACT-3′ R: 5′-AGGTCTCCAACATGCCTCTCTTC-3′
*Gapdh*	F: 5′-CTTCATTGACCTCAACTACATG-3′ R: 5′-CACAGTCTTCTGGGTGGCAGTG-3′
*H2a*	F: 5′-GGCCCGCGCCAAGGCCAAG-3′ R: 5′-CTCGTCGTTGCGGATGGCCAG-3
*Ppox*	F: 5′-CAGTTTTGCCCAGCGCCGCC-3′ R: 5′-GTCAGCCTCCAGACTGCTGTC-3′

## Discussion

The present study was initiated following the observation that mAb7G1-1 considered to immunoprecipitate specifically mFMRP, also reacts with Caprin1, another RNA-binding protein. We also showed that FMRP and Caprin1 interact independently of RNA and have defined the regions on each protein that are necessary for their interaction. During the course of the present study, Papoulas *et al.* [44] presented genetic evidence that in *Drosophila*, Caprin1 associates with dFMRP to regulate the cell cycle at the mid-blastula transition (MBT) during embryogenesis by mediating repression of maternal *Cyclin B* and activating the zygotic *Frühstant* mRNAs. These results confirm the observation that absence of Caprin1 results in defects in cellular proliferation [45]. The authors also reported that dFMRP, Caprin and eIF4G, a key regulator of translation initiation, were all three immunoprecipitated with an anti-dFMRP antibody, and suggested that the complex controls the translation machinery. Puzzling enough, immunoprecipitations were performed with dFMRP species detected at the top of the sucrose density gradient used to analyze polyribosomes, in fractions corresponding to the loading volume, far away from polyribosomes which were devoid of dFMRP.

While the physical interaction between FMRP and the RNA-binding protein LARK was reported to regulate eye development and circadian behavior in *Drosophila* [46], no functional relevance has yet been assigned for the interaction of FMRP with the RNA-binding proteins FXR1P, FXR2P, 82-FIP, NUFIP, MSP58 and now Caprin1 in mammals. Similarly to FMRP, Caprin1 has been shown to behave as a translation repressor [31]. In the case of FMRP, high levels of the protein lead to translational inhibition *in vitro* in the rabbit reticulocyte lysate system [47], [48]. In addition, in transfection assays, high levels of FMRP sequester mRNAs into granule-like structures resulting in repression of reporter genes [34]. On the other hand, FMRP functioning as an activator of translation has been documented only recently [49], [50]. The two scenario of FMRP being a translation repressor or activator might be reconciled if we envision that functions of FMRP depends on its levels which might vary in subcellular compartments [51]. Indeed neurons contain autonomous distinct subcellular micro-domains where translation takes place and mRNAs that have to be transported at these sites have to be embedded in a compact ribonucleoprotein structure (or granule) coupled with motor proteins allowing transport to distances far away from the soma [23], [28], [52], [53].

One of the most striking observations in the present study is that Caprin1 binds FMRP in a region that corresponds to the NES domain. The sequence QLRLERLQID necessary for FMRP to exit from the nucleus [54] lies precisely between amino acids 422 and 439 where Caprin1 binds FMRP according to our pulldown assays (Figure 3C). This strongly suggests two scenarios: 1) once FMRP exits from the nucleus, it can accept Caprin1 as a partner in the 422–439 region, since the NES is not required once in the cytoplasm; 2) the FMRP species that bind Caprin1 are not intended to undertaken any nuclear-cytoplasm shuttling and thus assemble with mRNP in the cytoplasm. It is worth mentioning that the majority of FMRP lies in the cytoplasm, while only trace amounts have been detected in the nucleus [6], [55].

As an RNA-binding protein, Caprin1 has been reported to interact with *Cyclin D2*, *c-Myc* [43] and *CaMKIIα* [31], mRNAs. Our analyses using mAb7G1-1 to immunoprecipitate Caprin1 from the KO mouse, indeed show the presence of these mRNAs. It is reasonable to conclude that some mRNAs might be common targets of Caprin1 and FMRP. Whether the two proteins bind to the same RNA molecule or that they function individually is not known. Consistent with our observations that mAb7G1-1 has an additional specificity for Caprin1, is the fact that RNA is immunoprecipitated from FMRP KO brain extracts (see Figure 2A in ref. 15). The substraction of immunoprecipitated RNA from KO from the WT-IP as was done by Brown *et al.* [15] might have introduced a misestimation of the levels and the nature of FMRP RNA targets in the case of mRNAs common to FMRP and Caprin1.

We also have clarified the observation that high salt treatment is required to allow immunoprecipitation of FMRP. It seems that FMRP is embedded in a structure that is not available neither to the monoclonal antibody 7G1-1 nor to the chicken IgY #C10 (as well as to other IgY from the same generation; unpublished results). Approximately 50 to 70% of FMRP present on polyribosomes are removed by high salt treatments and behave as free proteins or protein complexes. It is well established that in addition to its RNA-binding properties, FMRP also behave as a protein-protein adaptor to form homo-and hetero-dimers [56]. Whether these non-RNA binding species function as repressors or activators of translation is not known. The remaining 30 to 50% of FMRP was constantly found associated with polyribosomes and we propose that they represent the *bone fide* RNA-binding molecules.

Recent results from Darnell *et al.* [19] revealed an unexpected mode of FMRP-RNA interaction. No specific RNA motifs were identified by *in vitro* FMRP-RNA selection experiments and it seems that FMRP lays all over the coding sequences of the target mRNAs studied. In addition, previous studies have shown that the RGG box, thus the binding site to the G-quadruplex, is not essential for total FMRP to associate with polyribosome [21], [57], [58]. Although highly speculative, we propose that FMRP might function at two different levels : FMRP species that do interact with RNA and a second species that are protein adaptors interacting with different RNA-binding proteins in contact with RNA. Post-translation modifications might govern the fate and functions of FMRP towards these two avenues. As a working hypothesis, we propose that the high salt treatment, as used in the present report, removes the FMRP sheath from stalled polyribosomes leaving in place the FMRP species that have high affinity to mRNA targets.

In summary, we have shown that Caprin1 is a new FMRP interactor. Similarly to FMRP, Caprin1 is able to bind mRNA and is associated with the cytoplasmic translation apparatus and in trafficking granules in dendrites. Whether Caprin1 binds to FMRP to regulate its conformation (or vice versa) or has a synergistic effect, such as reported for FXR1P and FMRP [59], remains to be shown. As the world of RNA-binding proteins is expanding, understanding the role of Caprin1 and of other RNA-binding FMRP interactors, will be essential to unravel the functions altered by lack of *FMR1* expression in the fragile X syndrome. Since the absence of both FMRP and Caprin1 have been reported to induce dendrite dysmorphogenesis, it will be highly interesting to generate *Fmr1/Caprin1* double knockout mice to study whether *Fmr1* and *Caprin1* gene products interact or complement with each other. Finally, since mAb7G1-1 does not react with human FMRP, we consider it as an excellent monoclonal antibody to study Caprin1 in human cells.

## Materials and Methods

### Ethics Statement

Mice were bred in our animal facility and treated following the guidelines of the Canadian Council on Animal Care. This study has been approved by the University Laval ethics committee.

### Cell Cultures and Animals

HeLa S_3_ and NIH 3T3 cell lines were purchased from ATCC, and STEK *Fmr1* ^−/−^ KO cell line was established as described previously [34]. Cells were propagated and maintained in DMEM supplemented with 10% FBS and antibiotics (100 units/ml penicillin, 50 mg/ml streptomycin). Primary neuron cultures were prepared from neonatal rat hippocampi as described [60]. Wild-type C57BL/6J and *Fmr1* ^−/−^ KO2 strains [32], obtained from David Nelson, Baylor College of Medicine, Texas, USA, were bred in our animal facility.

### Protein Studies

#### Antibodies

IgY from egg yolks were purified using the Eggcellent^TM^ chicken IgY purification kit from Pierce. mAb7G1-1 [15] was affinity purified from the hybridoma supernatants using mFMRP immobilized on nitrocellulose membranes and the non-adsorbed remaining supernatant considered as IgG depleted. Immunoblot analyses were performed using hybridoma supernatants from mAb1C3 [5] and mAb7G1-1 [15] to FMRP, affinity purified rabbit IgG directed against Caprin1 were from [30], and Proteintech Group, affinity purified rabbit anti Ago2 (Cell Signaling), rabbit polyclonal anti-PABP (Cell Signaling), rabbit anti-L7 ribosomal protein (Novus Biologicals), rabbit anti-S6 ribosomal protein (Cell Signaling). Other antibodies used but not shown were rabbit anti-H3ABC, mAb14C8 anti-H2b and mAbCC3 anti-RNA polymerase II (obtained from Jacques Côté, Robert Tanguay and Michel Vincent, respectively). Detection of bound antibodies was performed with HRP-coupled secondary antibodies followed by ECL reaction.

For simultaneous double-immunoblot analyses, the Odyssey Infrared Imaging System from LI-COR was used. A mixture of mAb7G1-1 and rabbit antibodies against Ago2 was reacted with the membranes and revealed simultaneously with anti-mouse IR-Dye 800 (green) and anti-rabbit ID-Dye 700 (red) secondary antibodies (LI-COR). The two colors were imaged simultaneously in a single scan.

#### Immunofluorescence and histochemical analyses

Brain sections and hippocampal neurons grown on coverslips were processed as described [22], [23]. Samples were mounted in Prolong Gold medium (Invitrogen). Images were captured using a Zeiss LSM 510 confocal microscope and a 63×1.4 NA objective and analysed using the MetaMorph® Software.

#### Polyribosome preparation and analysis

Total brain polyribosomes were prepared from 10 days old wild-type C57BL/6J and *Fmr1* ^−/−^ KO2 mice, and analyzed as described [7]. For prolonged sedimentation in sucrose density gradients, samples were centrifuged at 34 000 rpm (198,045 g) for 23 hours in a SW41 rotor, and S values calculated using *E.coli* total RNA. Polyribosomes washed with 150 mM or 400 mM NaCl were concentrated by ultracentrifugation at 54 000 rpm (392,540 g) for 2 hours in 0.8 ml polyallomer tubes fitted in the Beckman SW 60 Ti bucket using a Delrin adapter (Seton, CA).

#### Immunoprecipitation

Affinity purified mAb7G1-1, IgY#C10 and affinity purified anti-Caprin1 were used in immunoprecipitations assays. For protein studies, immunoprecipitations of mFMRP were performed according to the protocol of Brown *et al.* [15] with the exception that BSA was omitted from all solutions. For RNA analyses, we applied the Brown protocol with no modifications.

#### Proteomics analyses

Analyses were performed at the McGill University-Genome Québec Innovation Centre facility (Montréal). Samples were run on SDS-PAGE, the gels were stained with Coomassie blue, scanned, and analyzed using the IMAGEMASTER software (Amersham Pharmacia). Gel slices were excised, proteins were *in situ* digested with trypsin, and the resulting tryptic peptides analyzed by mass spectrometry. Obtained sequences were interpreted with the Scaffold 3 Search program.

#### GST-pulldown assays

Coding sequences for *FMR1* iso7 and *Caprin1* were cloned into pGex-4T-1 (Amersham). Fusion proteins were expressed in BL21(DE3) *E.coli* strain (Stratagene) grown in liquid LB until OD≥1 and induced overnight with 1 mM IPTG. Bacteria were then collected and the expressed fusion proteins purified in non-denaturing conditions on glutathione-Sepharose beads, according to the manufacturers’ protocol (Amersham Pharmacia Biotech). Fusion protein was eluted from the beads with 10 mM reduced glutathione in 50 mM Tris-HCl (pH 8.0). Protein yields were estimated by Coomassie staining using as standard different concentrations of BSA ranging from 0.2 to 1 µg/µl. FMRP and Caprin1 and their truncated variants (see below cDNAs contructs) were produced by *in vitro* transcription-translation in rabbit reticulocyte system in the presence of [^35^S]-methionine (Amersham) according to the manufacturer’s instruction (Promega, Madison, WI). 5 µl of *in vitro*-translated proteins were mixed either with 1 µg of GST-FMRP or with GST-Caprin1 bound to beads and incubated 2 hr, at room temperature, under constant rotation in 500 µl of pulldown buffer (10 mM Tris pH 7.4, 150 mM NaCl, 0.5% NP40, Protease Inhibitor Cocktail, Roche). Beads were collected by spinning (5,000 rpm, at room temperature for 2 min.) and washed 4 times with the pulldown buffer. Final wash was removed and beads were resuspended in 50 µl of SDS-sample buffer. One third of the sample was loaded on a 7.5% SDS-PAGE. Gels were dried and exposed to Super RX films (Fuji).

### cDNA Contructs

All *FMR1* constructs used in the present study were described elsewhere [21], [22]. For *CAPRIN1* constructs, pGEX-*hCAPRIN1* plasmid [30] was digested by BamHI, and the resulting 2142-pb fragment subcloned into the pTL1 vector to generate pTL1-*hCAPRIN1*. Other constructs were generated by digestion of the pGex-*hCAPRIN1* by EcoR1/BamH1, and by HindIII/Pst1 and the fragments subcloned into pTL1 plamids to generate pTL1-*hCAPRIN1* (1–189) and pTL1-*hCAPRIN1* (75–267), respectively. For the other constructs, pTL1-*hCAPRIN1* plasmid was used as a template for PCR using as a standard the primer the T7 oligonucleotide forward 5′-TAATACGACTCACTATAGGG -3′. *CAPRIN1 (1–146)* was generated using reverse 440R: 5′-CGTTTCTGTTCAGCTTCTTCTC-3′; *CAPRIN1 (1–203)* with reverse 619R: 5′-CATTCAACCTCAAGCTCATGTC-3′; *CAPRIN11 (1–271)* with reverse 813R: 5′- TCAACTGCAGGTGCTGAGG-3′; *CAPRIN1 (1–349)* with reverse 1047R: 5′-ATTCACTGGAGTCAAAGAGTGG-3′; *CAPRIN1 (1–436)* with reverse 1308R : 5′-ATTCAAAGGAACCTGTGTCGCT-3′; *CAPRIN1 (1–498)* with reverse 1494R : 5′-ATTCCCAGCCTGAAATACTTGA-3′; and *CAPRIN1 (1–600)* with reverse 1799R: 5′-ATTGGAAATCCAGTGTTCTGCTGAG-3′.

To generate deletion mutants, pTL1-*hCAPRIN1* plasmid was used as template for PCR-mediated plasmid DNA deletion method. Primers were constructed to amplify the entire sequence of the plasmid except for the specific region that is to be deleted. The 5′ ends of the primers include the cutting sequence of the restriction enzyme EcoRI or PstI with one adjacent base at the 5′ and 3′ ends to facilitate the digestion close to the end of DNA regions. The sequences of the primers synthesized were:

Δ207–267∶5′-CAGCACCTGCAGTTGAAGA-3′, 5′- AACTGCAGGTTCCACATACAGGT-3;

Δ245–267∶5′-CAGCACCTGCAGTTGAAGA-3′, 5′- AACTGCAGGACTGAAAAACACGC-3′;

Δ230–267∶5′- AACTGCAGGTTCCACATACAGGT-3 , 5′-AACTGCAGTTTTCCTTCCCTTCC-3;

Δ215–267∶5′-CAGCACCTGCAGTTGAAGA-3′, 5′-AACTGCAGTGAATGGAGGCATGT-3′;

Δ186–216∶5′- CTTATAGAATTCATCCAAC-3′, 5′-GATGAATTCGTGGGACCTGCTGGA-3′.

### cDNA Synthesis, RT-PCR, and LightCycler Real-Time PCR

RNA immunoprecipitated by mAb7G1-1, IgY#C10 and anti-Caprin1 were extracted using the Trizol reagents according to the manufacturer’s protocol (Invitrogen). Reverse transcription using random poly dT primers (Invitrogen) and Sensiscript (Quiagen) was performed on 1 µg of RNA and one fifth of the resulting reaction was used for PCRs with 100 nM of the oligonucleotides primers listed in Table 1. PCR were performed by initial denaturation at 95°C for 5 min, followed by 35 cycles of denaturation at 95°C for 10 sec, annealing for 20 sec at 65°C, extension at 72°C for 30 sec and a final extension step at 72°C for 5 min. Amplified DNA fragments were fractionated on 1% agarose gels and stained with ethidium bromide.

Real-time PCR was performed with a LightCycler (Roche) using SYBR green chemistry (Ambion). The ratio and levels of different generated samples were calculated using the Light Cycler Data Analysis Software (Roche Molecular Biochemicals).

For analyses of mRNA present in polyribosomes, fractions obtained from sucrose gradients were extracted using the Trizol reagents. The purified RNA was subjected to reverse transcription and qPCR as detailed above. For each mRNA studied, normalization to three internal controls (*Gapdh, H2A and Ppox*) was performed and polyribosome fractions were plotted as percentages of the total amount of the mRNA in the collected fractions. All analyses were performed in triplicates.

## Supporting Information

Figure S1 **Sedimentation of FXR1P and FXR2P in presence of 150 and 400 mM NaCl.** For details see Figure 6 in the main text.
(TIF)
