# Correction: Fragile Mental Retardation Protein Interacts with the RNA-Binding Protein Caprin1 in Neuronal RiboNucleoProtein Complexes

**DOI:** 10.1371/annotation/05374d07-34cf-483f-80f4-ec87374cbeb6

**Published:** 2012-09-27

**Authors:** Rachid El Fatimy, Sandra Tremblay, Alain Y. Dury, Samuel Solomon, Paul De Koninck, John W. Schrader, Edouard W. Khandjian

There was a typographical error in the title and citation. The correct title is, "Fragile X Mental Retardation Protein Interacts with the RNA-Binding Protein Caprin1 in Neuronal RiboNucleoProtein Complexes" and the correct citation is:

El Fatimy R, Tremblay S, Dury AY, Solomon S, De Koninck P, et al. (2012) Fragile X Mental Retardation Protein Interacts with the RNA-Binding Protein Caprin1 in Neuronal RiboNucleoProtein Complexes. PLoS ONE 7(6): e39338. doi:10.1371/journal.pone.0039338 

