# Supplementary figures and images for: Fragile Mental Retardation Protein Interacts with the RNA-Binding Protein Caprin1 in Neuronal RiboNucleoProtein Complexes
Source: PLoS One. 2012 Jun 21;7(6):e39338. doi: 10.1371/journal.pone.0039338 (PMC3380850; doi:10.1371/journal.pone.0039338)

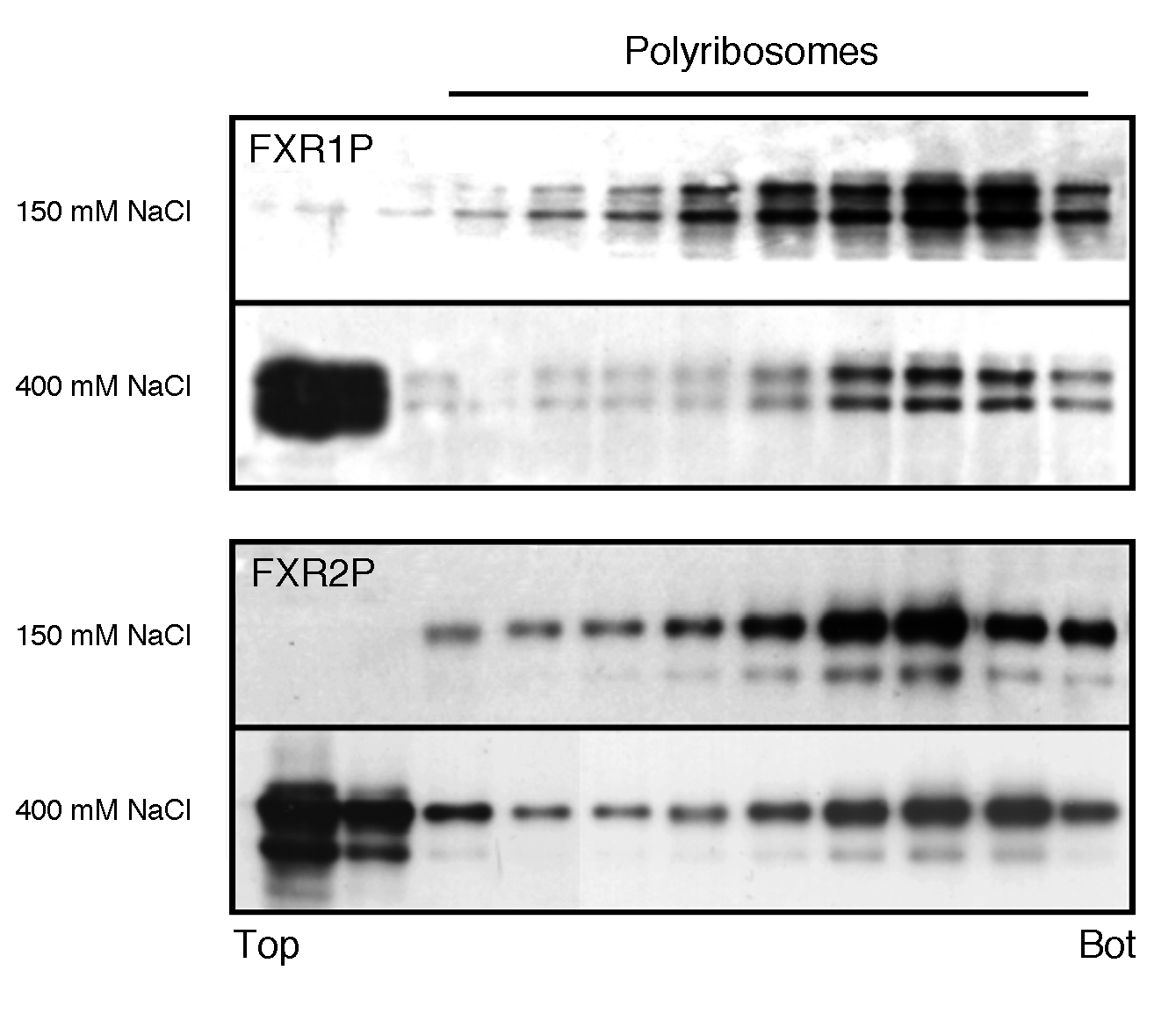

Supplement: Figure S1 — Sedimentation of FXR1P and FXR2P in presence of 150 and 400 mM NaCl. For details see Figure 6 in the main text. (TIF) [file pone.0039338.s001.tif]
